# Advances in chronic subdural hematoma and membrane imaging

**DOI:** 10.3389/fneur.2024.1366238

**Published:** 2024-04-25

**Authors:** Huanwen Chen, Marco Colasurdo, Ajay Malhotra, Dheeraj Gandhi, Uttam K. Bodanapally

**Affiliations:** ^1^National Institute of Neurological Disorders and Stroke, National Institutes of Health, Bethesda, MD, United States; ^2^Department of Neurology, MedStar Georgetown University Hospital, Washington, DC, United States; ^3^Department of Interventional Radiology, Oregon Health & Science University, Portland, OR, United States; ^4^Department of Radiology, Yale New Haven Hospital, New Haven, CT, United States; ^5^Department of Radiology, University of Maryland Medical Center, Baltimore, MD, United States; ^6^Department of Neurology, University of Maryland Medical Center, Baltimore, MD, United States; ^7^Department of Neurosurgery, University of Maryland Medical Center, Baltimore, MD, United States

**Keywords:** chronic subdural hematoma (cSDH), dual energy (CT), neurosurgery, embolization (therapeutic), neuroimaging (anatomic and functional)

## Abstract

Chronic subdural hematoma (cSDH) is projected to become the most common cranial neurosurgical disease by 2030. Despite medical and surgical management, recurrence rates remain high. Recently, middle meningeal artery embolization (MMAE) has emerged as a promising treatment; however, determinants of disease recurrence are not well understood, and developing novel radiographic biomarkers to assess hematomas and cSDH membranes remains an active area of research. In this narrative review, we summarize the current state-of-the-art for subdural hematoma and membrane imaging and discuss the potential role of MR and dual-energy CT imaging in predicting cSDH recurrence, surgical planning, and selecting patients for embolization treatment.

## Introduction

Chronic subdural hematoma (cSDH) is a common neurosurgical condition that has been growing in incidence (1, 2), likely due to the aging global population and increased use of antithrombotic medications. By 2030, the incidence of cSDH is expected to surpass that of intracranial tumors to become the most common cranial neurosurgical disease (3). The clinical and radiographic presentation of cSDH is highly heterogeneous, and conventional treatment modalities result in high rates of disease recurrence (4). In recent years, middle meningeal artery embolization (MMAE) has shown promise as a surgical adjunct or standalone treatment to prevent cSDH recurrence (5), and multiple large, randomized trials are currently underway. In this review, we highlight the current landscape of cSDH diagnosis and treatment, with a particular focus on imaging biomarkers that may be valuable in guiding patient selection for cSDH treatments.

## Epidemiology

The overall population incidence of cSDH has been reported to be approximately 10 per 100,000 persons in the general population and as high as 100 per 100,000 persons among the elderly population (6). Over time, cSDH incidence has grown, and its incidence is expected to continue increasing over the next two decades (1, 2). With conventional management, cSDH has a high rate of recurrence (4). Overall, patients with cSDH have a lower median survival time compared to non-cSDH patients with comparable health comorbidities (7). Once diagnosed, cSDH is associated with an excess mortality risk for up to 20 years (8). Whether these differences in patient outcomes are due to cSDHs themselves or associated comorbidities that may have facilitated the formation of cSDH (e.g., kidney disease, liver disease, cancer, or anticoagulation use) is unclear, as are details regarding neurological prognosis due to the heterogeneity of presenting symptoms.

On a societal level, cSDH incurs a significant financial burden. In a study of the National Inpatient Sample of hospitalizations in the United States (9), the median cost of hospitalization was $20,341 for non-surgical patients and $35,366 for patients requiring surgery. The total number of hospitalizations for cSDH in the United States is projected to be as many as 60,000 per year by 2030 (3), which could reflect a direct healthcare cost of nearly 2 billion dollars per year.

## Pathophysiology and subdural membranes

The pathogenesis of cSDHs, particularly their relationship with head trauma, is not well understood. On the one hand, some cSDH patients start with traumatic acute SDHs that persist and “mature” into a chronic state. On the other hand, a majority of cSDHs arise as a “*de novo”* disease following a minor head injury without a prior acute SDH stage (10). Moreover, many cSDH patients do not have a clear history of trauma. Regardless of the inciting event, it is believed that cSDH formation is initiated by an injury and irritation of the dural border cell layer and is considered to be an angiogenic and chronic inflammatory disease. Minor trauma to the dural border cell layer triggers a variety of reparative, angiogenic, and inflammatory factors similar to wound healing. The resultant granulation tissue organizes into (neo) membranes. The membranes consist of a thick external membrane that fully develops in 1 week and a thin internal membrane that develops in 3 weeks. The external membrane houses leaky, immature capillaries formed by angiogenesis and is considered crucial in the development and growth of cSDH. The immature capillaries are abnormally permeable due to their large gaps and sparse basal membrane. This allows for the continuous exudation of erythrocytes, leukocytes, and plasma into the subdural space, leading to gradual hematoma expansion. The fragile immature capillaries in the external membrane rupture intermittently, resulting in foci of hyperdense blood within cSDH that are seen on CT. Over time, cSDH volume accumulates, and the resulting mass effect can present with neurological symptoms. The external membrane is histologically classified into four types based on the maturity and intensity of the inflammatory reaction (11, 12):

- Type 1: Non-inflammatory. This type of membrane contains immature fibroblasts and collagen fibers, with minimal cell infiltration and neo-capillaries.
- Type 2: Inflammatory. This type of membrane is associated with marked inflammatory cell infiltration and neo-vascularization.
- Type 3: Hemorrhagic-inflammatory. This type of membrane has multiple layers associated with inflammation and many new vessels along the sides of the hematoma cavity, with hemorrhage into the membrane.
- Type 4: Scar-inflammatory. This type features inflammatory cell infiltration, neo-vascularization, and hemorrhage in the outer membrane of cicatricial tissue.

**Figure 1:**
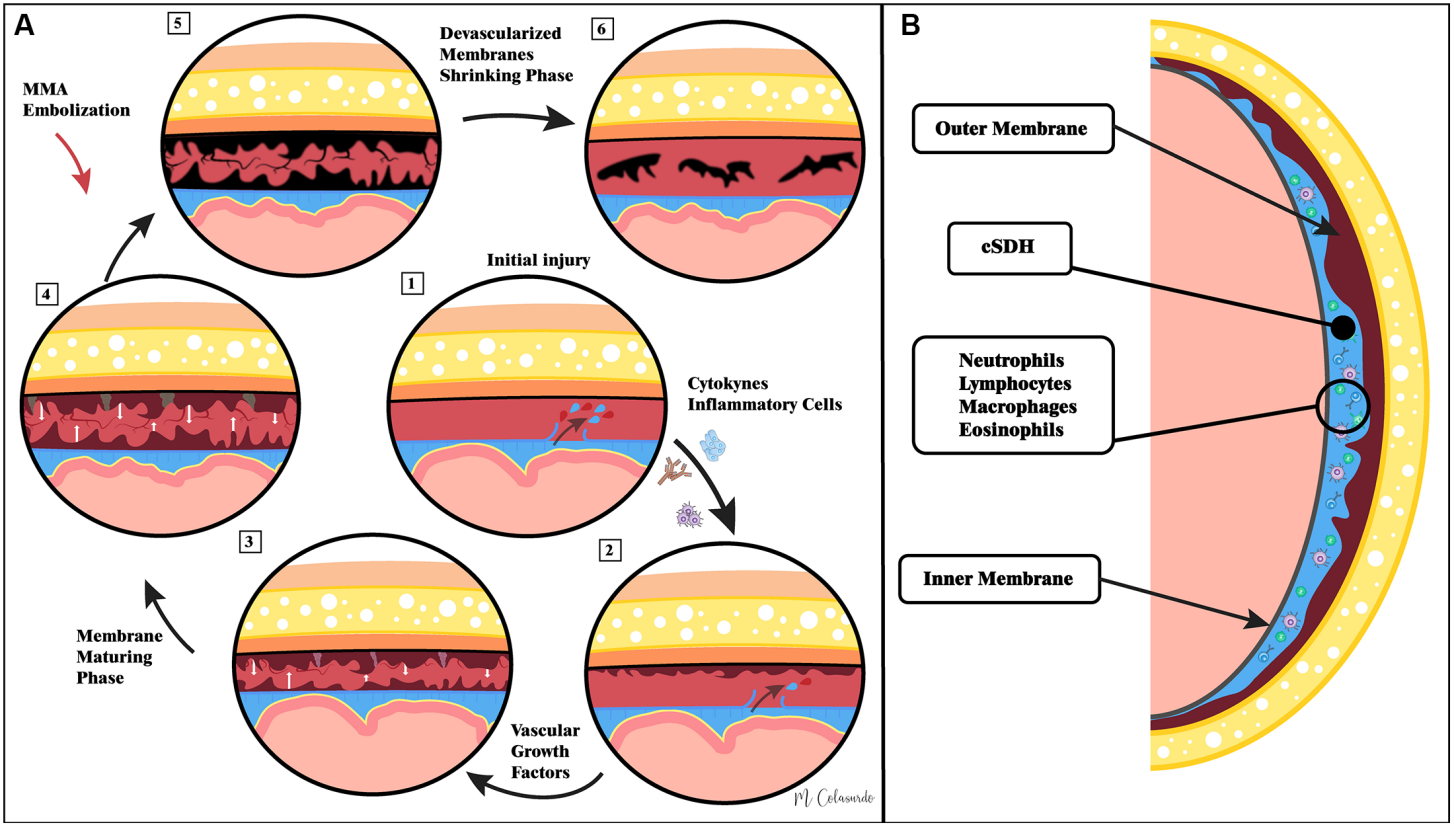
Illustrations of the current understanding of cSDH pathophysiology as well as the hypothesized role of middle meningeal artery embolization. Panel **(A)** demonstrates that pathophysiological events in cSDH formation and resolution following middle meningeal artery embolization. Panel **(B)** demonstrates the macro-anatomy of cSDHs.

The pathophysiology of cSDHs is depicted pictorially in Figure 1. Further details regarding the pathophysiology of cSDHs are reviewed elsewhere (13, 14).

## Conventional treatment modalities

Conventionally, cSDHs are either managed by surgical evacuation (for larger and more severely symptomatic cSDHs) or conservative medical management. While some cSDHs resolve without surgical intervention, many conservatively managed patients will go on to require eventual surgical drainage (15). Surgical procedures are usually required for patients presenting with neurological symptoms. Surgical methods range from burr-holes or twist-drill hole evacuation, with or without adjunct MMAE to craniotomy with membranectomy. Surgical evacuation can rapidly resolve mass effects and alleviate clinical symptoms; however, it carries high recurrence rates of up to 20% (4), suggesting that drainage alone is insufficient in addressing the chronic and recurrent nature of the cSDH disease. Furthermore, attempting an evacuation is bound to fail in hematomas consisting exclusively of solid membranes. Large craniotomies with membranectomy may yield lower rates of recurrence (16) due to the removal of leaky cSDH membranes; however, this major procedure carries substantial morbidity and mortality risk, and may not be appropriate for all cSDH patients, as many of whom are older and have comorbid coagulopathies (17).

Pharmacological treatments for cSDH are currently limited. Dexamethasone, an anti-inflammatory agent, has been extensively explored for cSDH given that aberrant inflammatory responses are thought to underlie cSDH pathophysiology; however, its efficacy data are mixed and overall do not support its routine use (18, 19). Atorvastatin has shown some promise in a phase II clinical trial, where it facilitated hematoma resorption and potentially reduced the need for eventual surgery by 50% among non-surgical cSDH patients (20). Larger trials are needed to further confirm the efficacy of atorvastatin for the treatment of cSDH. Other agents, such as tranexamic acid (pro-coagulant) and bevacizumab (anti-angiogenic agent), are also being investigated (21–23).

## Architectural classification and diagnostic criteria on non-contrast CT

Chronic SDHs commonly occur along the cerebral convexities, cranial base, and/or interhemispheric fissure, and their locations vary. The radiographic appearances of cSDHs can also be complex, ranging from iso- to hypodense, homogeneous to mixed densities, and single compartment to loculated.

Given their highly variable radiographic appearances, cSDHs do not have standardized diagnostic criteria. One accepted definition is the presence of predominantly iso- or hypodense subdural fluid collections, which is associated with the chronicity of blood products and may distinguish cSDHs from acute traumatic SDHs (18, 19). This definition, however, has limitations, as cSDHs can be difficult to distinguish from subdural hygromas based on radiodensity alone (24). Clinical diagnostic criteria are also challenging to establish, as patients present with a variety of focal neurological symptoms such as weakness, gait instability, and seizures. Given that clinical onset can be insidious, a time criterion for chronicity has limited value in a real-world setting. Surgical exploration can yield insight into the composition of cSDH fluids; however, not all patients will require or are appropriate for surgery. Thus, more sophisticated non-invasive imaging tools are needed to establish more precise criteria for identifying and diagnosing cSDHs.

Measurements of cSDHs are not standardized. Many clinicians and treatment trials use axial cuts on CT to determine maximal cSDH thickness. However, this measurement can be exaggerated due to the curvature of the cranium, especially near the vertex for cSDHs along the cerebral convexities. The mid-line shift is also a commonly used metric; however, it does not provide direct volumetric information regarding cSDHs. Other methods for measurements include subdural width at the center slice, corrected width, and cSDH length. Given the heterogeneity of cSDH morphology, geometry, and location, volumetric analysis likely provides more accurate information regarding cSDH size; however, imaging segmentation of cSDHs can be time- and resource-intensive and is not routinely employed in clinical practice. Artificial intelligence applications are available (25), and they may be employed to allow for rapid quantification of cSDH volumes in clinical use.

The internal architecture of cSDHs can be complex, and multiple classification schemes exist. The most widely cited classification system was published by Nakaguchi et al. (26), and here, cSDHs are categorized into four types—homogenous, laminar, separated, and trabecular. The classification was mostly developed as a radiographic tool to characterize the natural progression of cSDHs and predict cSDH recurrence following treatment using non-contrast CT. Homogenous and laminar subtypes are thought to be “younger,” and the separated subtype is thought to be associated with chronicity. The associations of these subtypes with the risk of cSDH recurrence are discussed in a later section.

## Imaging of cSDH membranes

**Figure 2:**
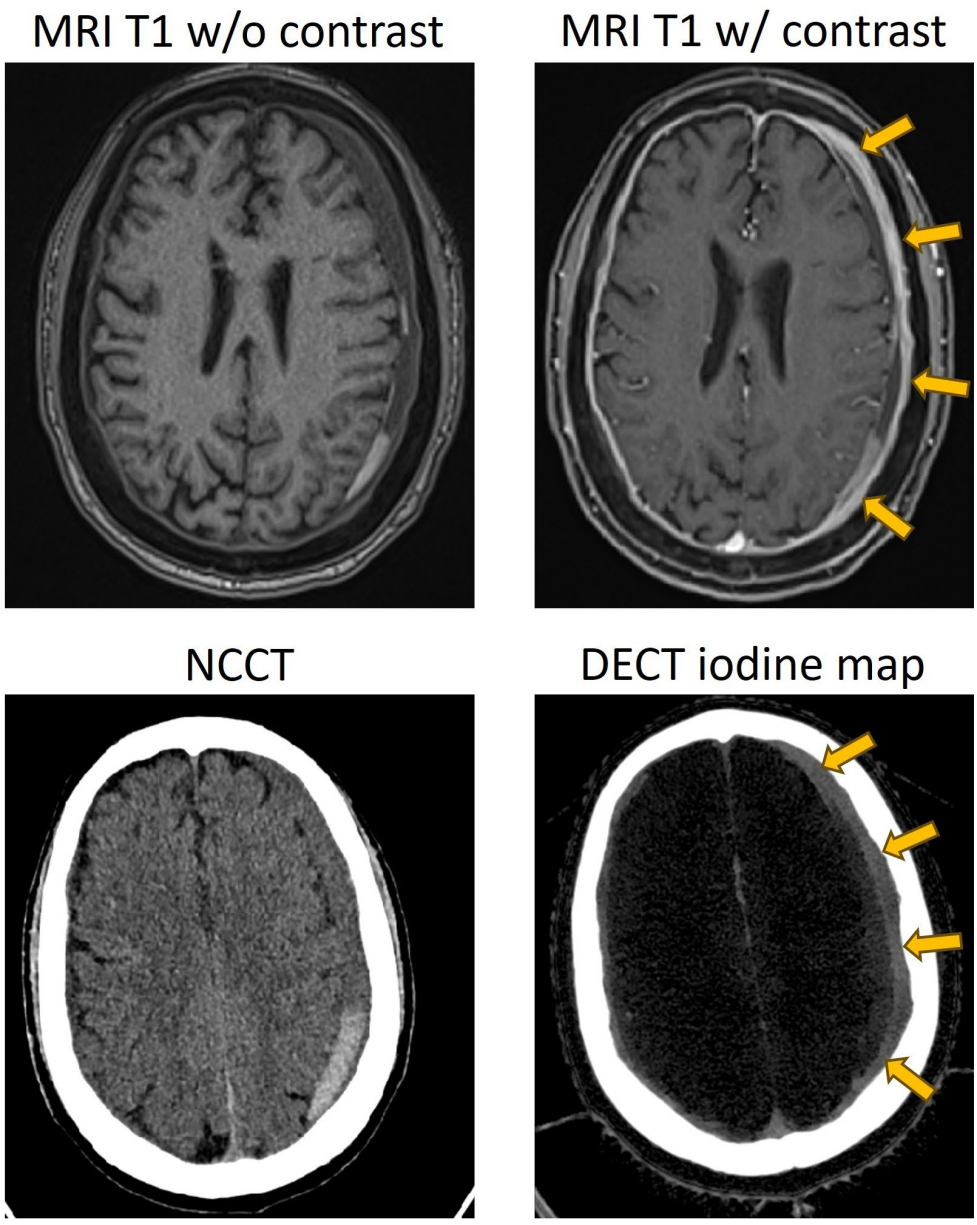
Representative case of a separated type of cSDH, with contrasted MR and dual-energy CT showing enhancement of the external membrane of a subdural hematoma (yellow arrows). NCCT, non-contrast CT; DECT, dual-energy CT.

**Figure 3:**
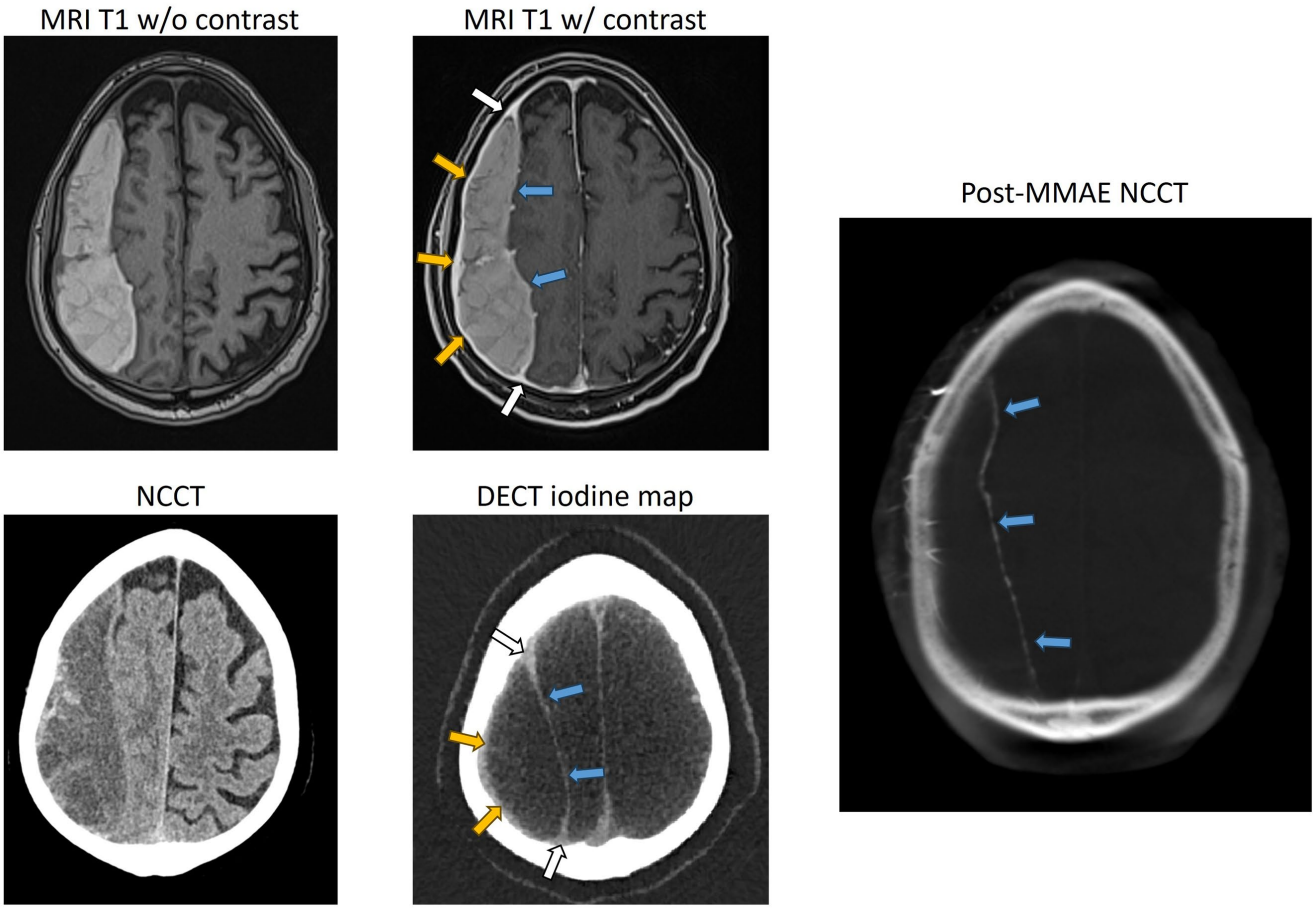
Representative case of a trabeculated cSDH, with contrasted MR, dual-energy CT, and DynaCT obtained during endovascular embolization showing the enhancement of the external membrane (yellow arrows), internal membrane (blue arrows), and “spandrel sign” (white arrows). Spandrel sign is defined as triangular thickened granulation tissue that forms the transition zone between the external and internal membranes. NCCT, non-contrast CT; DECT, dual-energy CT.

Imaging cSDH membranes and analyzing their morphology have always been challenging in clinical practice. Hence, the majority of patients are triaged and treated based on the imaging information and morphology of the hematoma that is obtained on a non-contrast head CT. However, for cSDHs that appear heterogeneous, compartmentalized, or septated, or for those that have recurred after treatment, membrane imaging by contrast MRI or contrast DECT (Figures 2, 3) may yield valuable information to guide treatment and is recommended (27, 28).

### Contrast MRI

Contrast-enhanced MRI has long been established as a non-invasive imaging modality for the demonstration of membranes and their morphology (29). Imaging of the membranes not only provides information about the morphology but also the extent of distribution over the cerebral lobes, thus determining the location and extent of craniotomy during surgical treatment (27). Incomplete resection of membranes, especially those that are in hard-to-reach sections over the inferior temporal or frontal lobes, is associated with recurrences (26). Hence, localization and assessment of the extent of membrane distribution over the cerebral hemispheres are important before embarking on a craniotomy. Contrast MRI has the additional capability of providing precise information about the liquid and solid components of cSDH, in addition to its ability to better delineate CT-isodense cSDH (30). Spreer et al. (29) observed enhanced external membranes in 16 out of the 17 contrast MRI scans and demonstrated that external membranes can be visualized in an overwhelming majority of patients, independent of the stage of hematoma (Figure 2). Enhancing internal membranes that were demonstrated in 9 out of the 17 scans were all in the late stages of hematoma (Figure 3). Contrast MRI also demonstrates the transition zones between the external and internal membranes that would manifest as triangular spandrel-like thickening (31). The transition zones are found to have abundant newly formed capillaries in histologic studies (32). Hence, meticulous and complete resection of transition zones during membranectomy is essential for reducing recurrences (27). In long-standing hematomas, progressive fibrosis reinforces the external membrane, transforming the hematoma into a completely solid structure (12, 33). Contrast MRI’s ability to identify hematomas consisting exclusively of solid membranes can help mitigate unnecessary burr-hole or twist-drill evacuation procedures as these treatments are invariably going to fail (30). Instead, craniotomy with membranectomy should the preferred surgical approach in patients with solid cSDH membranes. Non-contrast CT in such patients with solid membranes tends to demonstrate low-density areas mimicking the liquid component of hematoma (34).

### Dual-energy CT

Similar to contrast MRI, contrast dual-energy CT (DECT) facilitates the visualization and localization of membranes and helps assess the composition of hematomas by providing information about the liquid component of the hematoma. It also provides information about the thickness and complexity of the membranes, helping assess the maturity and grading of the membranes (28). It was demonstrated that iodine maps from 5-min delayed post-contrast DECT facilitate the segregation of membranes (Figures 2, 3). Iodine maps capture iodine and help in demonstrating the tissues that are enhanced with iodinated contrast, utilizing the concept of water-iodine base pairing in basis material decomposition (BMD) (35). Furthermore, the iodine map transforms and references all the remaining tissues that are not enhanced, including the brain parenchyma and hematoma, to water, providing the spectral contrast between the contrast-enhanced membranes and the adjacent tissues (35).

### Classification of membranes

**Figure 4:**
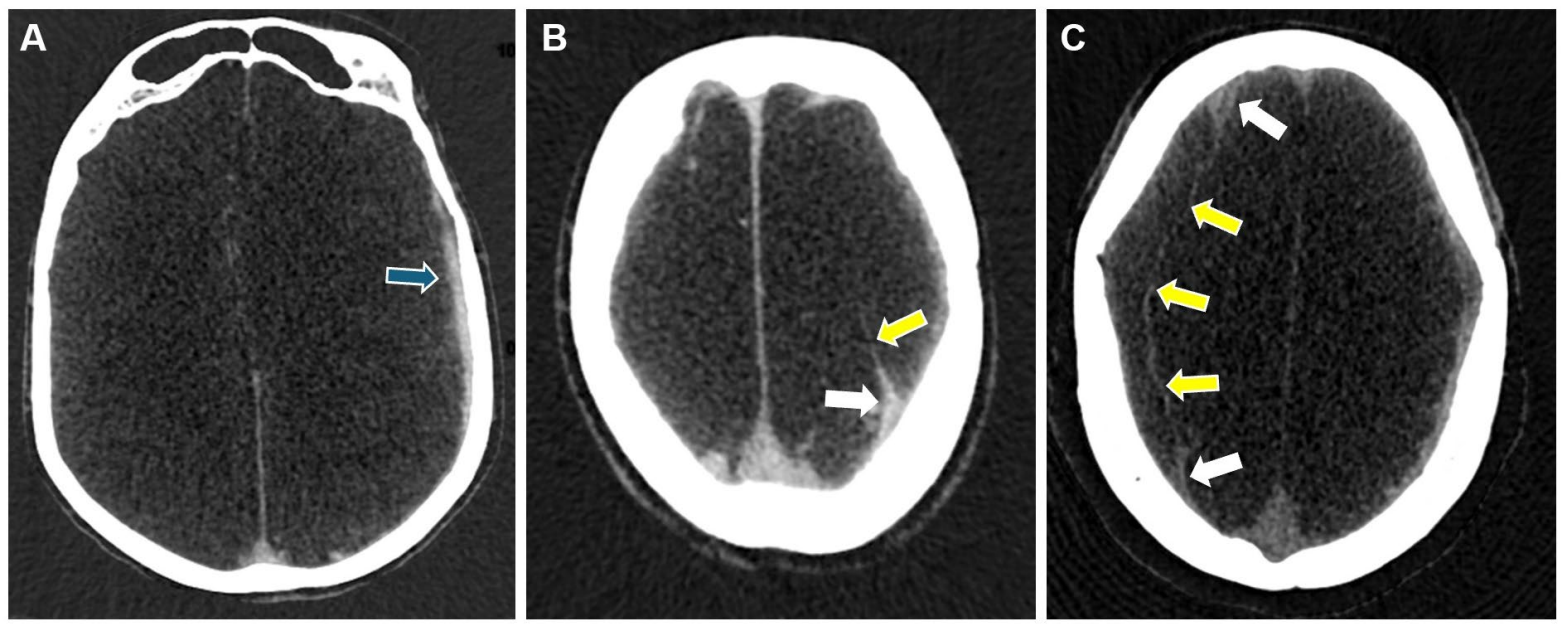
Representative images for cSDH membrane subtypes seen on DECT. Panel **(A)** shows a Grade I membrane with an enhanced external membrane (blue arrow). Panel **(B)** shows a Grade II membrane with an additional “spandrel sign” (white arrow) and a partially enhanced internal membrane (yellow arrow). Panel **(C)** shows a Grade III membrane with “spandrel signs” (white arrows) and a completely formed and enhanced internal membrane (yellow arrows).

Studies using contrast-enhanced MRI and DECT have shown progressive thickening of the external membrane, followed by the development of internal membranes in the late stage. Internal membrane enhancement on MRI and DECT was defined as advanced cSDH with mature membranes (28, 29). Based on the evolution of these changes in the membrane morphology, they are graded on DECT depending on the pattern of membrane enhancement: Grade I-enhanced external membrane, Grade II-early phase of internal membrane formation with “spandrel sign,” and Grade III-enhanced external membrane and completely formed internal membrane (28) (Figure 4). The formulated DECT membrane grades on DECT correlated with the degree of hyperdense component in the hematomas (36). Hematoma density has been reported to be associated with cSDH recurrence risk after burr-hole irrigation (37). Nakagawa et al. (38) used a similar grading system based on DynaCT images obtained during middle meningeal embolization. They demonstrated more frequent recurrences in patients with higher membrane grades.

### Functional assessment of cSDH membranes

Functional information obtained from the membranes is shown to correlate with recurrences after burr-hole evacuations. An invasive procedure involving the injection of technetium-99 m human serum albumin and measuring the radioactivity level in evacuated hematoma at the burr-hole site has shown that exudation rates from the membranes are an important predictor of hematoma recurrences and are correlated with hematoma size (39). The study showed a higher rate of exudation in hyperdense and mixed-density hematomas than in hypodense and isodense hematomas. Recently, it was shown that DECT after contrast administration has the ability to estimate membrane exudation by using the quantification of iodine leaks through the immature capillaries in the external membrane (36). DECT, by having two attenuation measurements for iodine at two different x-ray spectra, enables the mathematical conversion of attenuation data into estimates of iodine concentration (35). The capacity to quantitatively analyze leaked iodine concentration within the subdural hematoma can be used as a surrogate of membrane exudation and the magnitude of angiogenesis in membranes. The ability to visualize the membranes and measure the iodine concentration on DECT at the same time will help us understand the relation between the morphology and permeability of the membranes, given the dominant role played by the membranes in the origin and sustenance of cSDH. DECT exudation has shown a higher iodine leak in separated-stage hematomas (36). The total iodine leak increased with the chronological stage of the hematomas, reaching its maximum values at the separated stage and then decreasing in the trabecular type of hematoma (36). A high iodine leak in mature membranes supports the findings described by Nakaguchi et al. (26) and histological findings of increasing capillaries and inflammatory cells as the membrane matures to the separated stage. This is followed by a decrease in the trabecular stage, which is considered the resolution stage due to the fibrosis of the membranes. Studies also showed high recurrence rates in separated-type hematomas after burr-hole evacuation (37). Gadolinium contrast leakage can also be seen with contrast MRI; however, its impact on the disease course of cSDH is less clear.

Quantification of membrane exudation, visualization, and grading of the membranes can allow for the examination of longitudinal alterations of the membranes and may provide information about their susceptibility in terms of morphology and permeability changes after different forms of treatments (tranexamic acid, dexamethasone, MMAE, burr-hole washout, etc.). Analysis of these imaging markers and their responses to different treatments can give us a better understanding of the role played by membrane grades and their exudation on the recurrence rates and rate of hematoma volume reduction after specific treatments. Data from such studies should help tailor precision therapies based on imaging findings and provide a framework for better understanding the pathogenesis of CSDH that can later fit into emerging spectral photon-counting molecular CT imaging.

## Middle meningeal artery embolization

The middle meningeal artery (MMA) has long been known to provide vascular supply to the dura; thus, it is possible that restricting arterial blood supply by the endovascular embolization of the MMA may lead to the necrosis of subdural membranes and neo-vasculature, which are thought to be responsible for cSDH persistence and recurrence. As such, MMA embolization (MMAE) is hypothesized to be an effective adjunctive treatment to surgical evacuation to reduce the rates of future cSDH recurrence (Figure 1).

MMAE emerged as a promising treatment for cSDHs in 2018 when Ban et al. (5) reported that MMAE resulted in a strikingly low rate of recurrence (1.4%) compared to conventional management (27.5%). Since then, multiple studies have corroborated these findings, with a meta-analysis reporting a recurrence rate of under 5%, followed by MMAE, compared to more than 20% with conventional management (40). In addition to preventing recurrence, MMAE may also be effective in promoting cSDH resorption and resolution without surgical evacuation. In a small, randomized study of 46 patients, Ng et al. showed that adjunctive MMAE in addition to surgical treatment led to a significantly larger cSDH volume resorption at a median of 3 months (41). Catapano et al. also showed that MMAE led to significantly larger decreases in cSDH thicknesses at follow-up compared to conventional management (42). As such, MMAE could also be an effective standalone treatment that can be offered as a non-surgical alternative to patients with smaller hematoma volumes and mild symptoms (43, 44).

In February 2024, preliminary results from the EMBOLISE (45), MAGIC-MT (46), and STEM (47) trials were presented at the International Stroke Conference. All three trials investigated the role of adjunctive MMAE for the treatment of cSDH in addition to standard of care (surgery or observation). All three trials met their primary efficacy endpoints for cSDH recurrence and demonstrated excellent safety data for MMAE. Given these positive results, it is likely that MMAE will be incorporated into the standard of care for SDHs. The formal conclusion of these landmark trials as well as the final peer-review and publication of the results are now eagerly awaited. Other randomized trials, such as MEMBRANE (NCT04816591) and SWEMMA (NCT05267184), are also underway.

## Radiographic predictors for cSDH recurrence

While a substantial portion of cSDH patients experience hematoma recurrence following surgical evacuation, it is important to recognize that most cSDHs resolve after treatment. As such, identifying patients at high risk of surgical recurrence may help providers select patients for treatment modalities targeted at preventing disease recurrence, such as MMAE, regardless of the results of ongoing trials.

**Figure 5:**
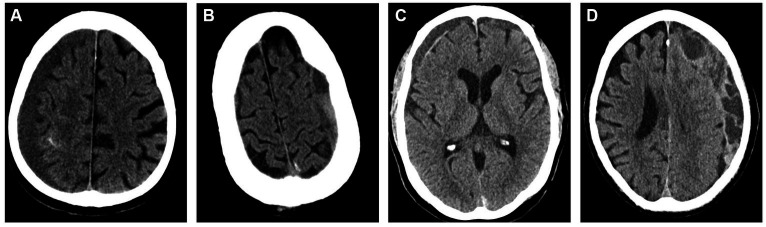
Representative images for cSDH subtypes based on the Nakaguchi classification system. Panel **(A)** – homogenous; Panel **(B)** – separated; Panel **(C)** – laminar, Panel **(D)** – trabeculated.

The heterogeneity of cSDH’s radiographic morphology has been extensively studied as a potential biomarker for disease recurrence. Multiple classification systems exist for characterizing the cSDH internal architecture. The most common scheme is the Nakaguchi classification system (26). In this study, cSDHs are categorized into four types–homogenous, laminar, separated, and trabecular. Homogenous and laminar (the early subtypes) are less likely to recur, while the separated hematoma (the later subtype) is more likely to recur after treatment. Data on outcomes following MMAE stratified by Nakaguchi cSDH subtypes corroborated these results, suggesting that the separate subtype was associated with slower hematoma resorption following MMAE. Trabecular subtypes were thought to be associated with resolving cSDHs with a low risk of recurrence. Representative images of the Nakaguchi classifications are presented in Figure 5. In a separate scheme, Nomura et al. (48) classified cSDHs by pattern of radiodensity (hyperdense, isodense, hypodense, mixed, or layered) and showed that the layered and mixed cSDHs were more likely to recur, while hypodense cSDHs were less likely to recur. Shimizu et al. (49) also demonstrated that gradation density hematomas are at higher risk of recurrence. More recently, Takei et al. (50) proposed modified criteria combining these classification systems into five subtypes—homogenous, gradation, laminar, separated, and trabecular—and showed that this modified system had higher interrater agreement than the Nakaguchi and Nomura classifications and that the gradation subtype was most strongly associated with cSDH recurrence. Research on radiographic predictors of cSDH recurrence remains an active area of investigation.

Given that leaky vessels housed within cSDH membranes are thought to underlie the pathophysiology of cSDH persistence and recurrence, membrane imaging is also of great interest for the prediction of cSDH recurrence. In a study of 40 cSDHs, Tanikawa et al. (51) showed that MR imaging can be used to visualize cSDH membranes and that patients with intrahematoma membranes were significantly more likely to recur following burr-hole drainage compared to craniotomy. On catheter angiogram, highly vascularized cSDH membranes may appear similar to tumor blushes with increased contrast staining during the capillary phase, and this too can be used as a predictor for recurrence risk.

Importantly, early studies showed that a high rate of protein exudation into cSDHs is associated with higher rates of cSDH recurrence (39). In a retrospective study of 27 patients with 29 cSDHs, Mureb showed that MMAE procedures (which involve copious amounts of intra-arterial iodinated contrast administration) led to enhancements of cSDHs and their membranes in all patients (52), suggesting that iodinated contrast enhancement of subdural membranes and leakage into hematomas may be a biomarker for underlying pathophysiology and possibly a predictor of recurrence risk. As previously discussed, DECT also enables the non-invasive and precise quantification of iodine leakage into cSDHs (28). This has been shown to be the highest in separated types of hematomas, which are associated with higher rates of cSDH recurrence after burr-hole evacuation and a slower rate of resolution after sole MMAE (53).

## Conclusion

cSDH is a common neurosurgical disease that is expected to rapidly grow in global incidence. Disease recurrence is common despite conventional management. MMAE has emerged as a promising treatment for preventing surgical recurrence, and clinical trials are currently underway. The development of radiographic markers for cSDH recurrence remains an active area of investigation, and DECT techniques are promising for the quantification of iodine leakage and may be leveraged toward improved patient selection algorithms for cSDH treatment.

## Author contributions

HC: Conceptualization, Writing – original draft. MC: Visualization, Writing – original draft. AM: Writing – review & editing. DG: Writing – review & editing. UB: Conceptualization, Project administration, Supervision, Writing – original draft.
